# Depression symptoms as longitudinal predictors of the psychological impact of COVID-19 pandemic in hypertensive patients

**DOI:** 10.1038/s41598-021-96165-2

**Published:** 2021-08-13

**Authors:** Marco D’Addario, Francesco Zanatta, Roberta Adorni, Andrea Greco, Francesco Fattirolli, Cristina Franzelli, Cristina Giannattasio, Patrizia Steca

**Affiliations:** 1 Department of Psychology, University of Milano-Bicocca, Milan, Italy; 2 Department of Human and Social Sciences, University of Bergamo, Bergamo, Italy; 3 Department of Experimental and Clinical Medicine, University of Florence, Florence, Italy; 4 Azienda Ospedaliero Universitaria Careggi, Florence, Italy; 5 Cardiac Rehabilitation Centre, CTO Hospital, Milan, Italy; 6 School of Medicine and Surgery, University of Milano-Bicocca, Milan, Italy; 7 Cardiology IV, “A. De Gasperis” Department, Ospedale Niguarda Ca’ Granda, Milan, Italy

**Keywords:** Risk factors, Human behaviour

## Abstract

COVID-19 has brought considerable changes and caused critical psychological responses, especially among frail populations. So far, researchers have explored the predictive effect of diverse factors on pandemic-related psychological distress, but none have focused on the impact of prior depression and anxiety symptomatology adopting an extended (10-year) longitudinal design. 105 patients aged over 60, affected by hypertension who participated in a previous longitudinal study were assessed through a follow-up telephone structured interview. The Hospital Anxiety and Depression Scale (HADS) and the Impact of Event Scale-Revised (IES-R) were used for assessing depression and anxiety symptoms and the psychological impact of COVID-19, respectively. Multiple linear regression analyses were conducted. At the assessment, participants did not report clinically relevant depression, anxiety, and psychological pandemic-related distress symptoms. However, significant mean differences between baseline and current follow-up evaluations for both depression and anxiety were found, reflecting a decrease in symptomatology over time (*p* < .001). Baseline depression symptoms (*β* = 1.483, *p* = .005) significantly predicted the psychological impact of COVID-19 after 10 years. Conversely, their decrease (*β* = −1.640, *p* < .001) and living with others (*β* = −7.274, *p* = .041) significantly contributed to lower psychological distress scores. Our findings provide insight into the predisposing influence of depressive symptoms on pandemic-related psychological distress ten years later. Preventive interventions and strategies considering these factors are needed to better pre-empt the severe mental consequences of the pandemic.

## Introduction

On 11th March 2020, the World Health Organization (WHO) officially declared the Coronavirus disease-2019 (COVID-19) outbreak as an event involving worldwide public health^1^. So far, over 150 million people have been infected in more than 200 countries and regions worldwide, and over 3.5 million deaths have been confirmed^2^. In particular, Italy was the first European country to report a case of COVID-19 and since the onset of the pandemic in China at the end of December 2019, it has been the country with the highest number of deaths in Europe so far^2^. In response to the rapid spread, immediate precautions and dispositions were adopted affecting people’s daily lives and causing considerable psychological strain. Indeed, not only does the pandemic constitute an unprecedented healthcare crisis, but also a challenging and overwhelming occurrence that has triggered widespread anxiety, depression, distress, insomnia, and fear in the general population^3,4^.

Such mental health disorders were shown to be attributable to various factors, including gender, social support, length of isolation, and specific experiences with COVID-19 infection^5,6^. Their severity was also shown to vary in relation to different health and social conditions^7^. As yet, the literature has explored the association of socio-demographic and health-related factors with individuals’ responses to the event and their likelihood of developing psychological disorders. For instance, recent studies^4,8,9^ have suggested a role of gender: women reported significantly higher psychological distress than men resulting in moderate anxiety levels. Moreover, older age and a medical history of chronic illness were related to higher levels of psychological distress, as they are acknowledged to be the most critical risk factors for developing more severe forms of COVID-19.

Also, other health-related disorders like hypertension were shown to be determinant. Hypertension represents one of the most common diseases worldwide causing mortality and disability, and it was shown to be associated with depression and anxiety symptoms^10,11^. Moreover, co-morbid hypertension and mental disorders are not only associated with a higher-risk for cardiovascular disease-related mortality^12^, but also with worse prognoses through the COVID-19 infection course^13,14^. Additionally, containment measures including social distancing and self-isolation had a further impact and negatively affected people’s well-being^5^. Prior studies have found that higher levels of restrictions due to lockdown measures were related to higher psychological distress, lower life satisfaction, and loneliness^15^ and that the latter strongly predicted depressive and anxiety symptomatology^16,17^. Consistently, stronger and prolonged social contact reduction and consequent stronger perceived changes in life due to quarantine were shown to be associated with poorer mental health and to contribute to psychiatric symptoms onset, too^18–20^.

Overall, COVID-19 has caused a considerable health emergency that, in turn, has provoked severe psychological responses requiring timely and urgent interventions^21^. Hence, special attention should be reserved to those populations with higher levels of frailty whose condition may represent a critical factor. As underscored in prior research, pre-existing conditions of depression and anxiety may further strengthen the effect of a stressful event^22–24^, and intense psychological distress was shown to be strongly related to poorer health outcomes and increased mortality risk, especially among populations affected by chronic conditions^25^. Although this association was prospectively explored, it provided relatively short-term insights (typically up to 1 year) and was not under pandemic circumstances. Following this line, the present study aimed at estimating to what degree depression and anxiety symptoms and their longitudinal change predicted the psychological impact of the COVID-19 pandemic in a cohort of frail older patients suffering from a non-communicable disease, namely arterial hypertension. Notably, the timeframe considered for the present longitudinal study was 10 years. Based on the evidence of recent literature on the associations among socio-demographic factors, depression, anxiety and psychological distress^7,26^, we expected that, controlling for socio-demographic factors, higher levels of depression and anxiety symptoms would longitudinally predict worse psychological responses related to the pandemic, shedding light on their predisposing influence over time.

## Results

### Participants’ general and COVID-19-related health conditions

The 92% of participants perceived good general health, although 23 of them reported a period of hospitalization after the last contact due to cardiovascular, pulmonary or oncological events. Most reported no COVID-19 contagion (83.7%) and others reported mild symptoms but did not undergo testing and, thus, did not receive a diagnosis (16.3%), while the 24.5% reported having relatives who had contracted the virus (Table 1).

**Table 1:** COVID-19-related health indicators of the study sample.

Variables	
**COVID-19 contagion**	
No	87(83.7%)
Mild symptoms, but no diagnosis	17(16.3%)
**Covid-19 contagion—relatives**	
No	77(75.5%)
Yes	25(24.5%)

### Depression, anxiety, and psychological distress

The mean scores of anxiety and depression symptoms, including the delta corresponding to the difference between the baseline and the current follow up, and scores on the psychological impact of COVID-19 are reported in Table 2. The paired-samples *t*-test showed significant mean differences for both anxiety (2.5 ± 3.9) and depression (1.2 ± 3.1) between the two timepoints reflecting a significant reduction in symptomatology after ten years (*p* < 0.001).

**Table 2:** Anxiety and depression, and the psychological impact of COVID-19 in study participants.

	Baseline	Follow-up	Δ*	*t*	*P* value	*d*
**HADS**						
Anxiety (range 0–21)	7.1 ± 3.7	4.6 ± 3.7	2.5 ± 3.9	6.369	< 0.001	0.62
Depression (range 0–21)	4.3 ± 3.0	3.1 ± 3.4	1.2 ± 3.1	3.786	< 0.001	0.37
IES-R (range 0–88)		18.1 ± 12.8				

Data are mean ± standard deviation.

*Delta was calculated from the difference between the baseline and follow-up mean scores of anxiety and depression.

α ≤ 0.05 indicates significant difference. Cohen’s d was reported as indicator of effect size from the paired samples *t*-test.

### Longitudinal prediction of COVID-19-related psychological distress

Moderate significant positive correlations were found between psychological distress and baseline anxiety (*r* = 0.317, *p* = 0.001) and psychological distress and baseline depression (*r* = 0.373, *p* < 0.001).

The multiple linear regression analyses showed significant simultaneous impacts on the perceived psychological distress related to COVID-19. Two models were analysed. In the first one, the living conditions (*β* = − 7.274, *p* = 0.041), and the depression symptoms measured at baseline (*β* = 1.483, *p* = 0.005) emerged as significant predictors, meaning that living with others mitigated the levels of psychological distress, while higher levels of depression contributed to higher outcome scores. Age, gender, employment status, time since hypertension diagnosis, and baseline anxiety provided no significant impact (Table 3). The model explained 21.1% of the variance and estimated a medium-large effect size (*f*^2^ = 0.27). Moreover, a significant regression equation was found (*F*[7, 88]  = 4.630, *p* < 0.001).

**Table 3:** Multiple linear impact of socio-demographic variables, and baseline anxiety and depression symptoms on IES-R (n = 96).

	*β*	*t*	95% CI		*P* value
Age	−0.246	−1.175	−0.662	0.170	0.243
Gender	−0.035	−0.014	−4.902	4.833	0.989
Living conditions	−7.274	−2.072	−14.250	−0.297	0.041
Employment status	4.920	1.769	−0.606	10.446	0.080
Time since hypertension diagnosis	−1.313	−0.460	−6.990	4.363	0.647
HADS Anxiety (baseline)	0.492	1.202	−0.321	1.306	0.232
HADS Depression (baseline)	1.483	2.905	0.468	2.498	0.005

Data are the unstandardized regression coefficients (*β*), the t-test value (*t*), and confidence interval (95%).

The second model confirmed the significant prediction of depression symptoms. Those measured at baseline (*β* = 2.261, *p* < 0.001) and their change over time (*β* = -1.640, *p* < 0.001) significantly predicted IES-R scores, meaning that higher levels of depression symptoms predicted higher levels of psychological distress after ten years and their decrease over time resulted in lower outcome scores. Neither the socio-demographic parameters nor anxiety (baseline and delta) emerged as significant independent variables (Table 4). A significant explained variance of 33.8% emerged from the model (*F*[9, 86] = 6.382, *p* < 0.001), and a large effect size (*f*^2^ = 0.51) was observed. A post-hoc power analysis was performed to estimate the achieved statistical power of the models. The analysis showed satisfactory results (first model, 1 − *β* = 0.97; second model, 1 − *β* = 0.99).

**Table 4:** Multiple linear impact of socio-demographic variables, baseline anxiety and depression symptoms, and their change after 10 years on IES-R (n = 96).

	*β*	*t*	95% CI		*P* value
Age	−0.277	−1.422	−0.665	0.111	0.159
Gender	1.036	0.457	−3.468	5.540	0.649
Living conditions	−3.971	−1.169	−10.722	2.780	0.245
Employment status	2.526	0.969	−2.657	7.709	0.335
Time since hypertension diagnosis	−1.043	−0.398	−6.248	4.161	0.691
HADS Anxiety (baseline)	0.579	1.270	−0.327	1.486	0.207
HADS Depression (baseline)	2.261	4.373	1.233	3.289	< 0.001
HADS Anxiety (Δ)	−0.151	−0.385	−0.931	0.629	0.701
HADS Depression (Δ)	−1.640	−3.765	−2.506	−0.774	< 0.001

Data are the unstandardized regression coefficients (*β*), the t-test value (*t*), and confidence interval (95%).

## Discussion

The present study aimed at estimating the longitudinal impact of depression and anxiety symptoms on the psychological response to the ongoing COVID-19 pandemic. Specifically, depression and anxiety symptomatology assessed at baseline and 10 years later were considered as possible predisposing factors determining the degree of psychological distress. The purpose was driven by prior evidence suggesting positive associations among these factors and showing that pre-existing conditions of depression and anxiety may further foster the effect of a stressful event, especially among populations characterized by frail conditions^22–24^. Consistently, a cohort of patients aged over 60 and affected by arterial hypertension were included.

Results show that on average the study sample reported neither clinically relevant depression and anxiety symptoms, nor severe rates of psychological distress. These results are not surprising if we consider that none of the interviewees had contracted COVID-19 and that the majority had no relatives who had been infected, thus avoiding a first-hand experience or direct contact with the virus. Although the pandemic has been generally recognized to provoke psychological effects, prior works have reported evidence of relevant differences between populations affected by the virus and those not. Accordingly, a recent meta-analysis showed that pandemic-affected populations provided a significantly higher prevalence of depression, anxiety, insomnia, psychological distress, and post-traumatic stress disorder (PTSD) when compared to the general population^26^. Moreover, it was shown globally that during the pandemic older adults reported overall lower levels of depression, anxiety, and stress than younger age groups^27^. At baseline, the study sample reported higher mean scores for depression and anxiety, and a significant decrease in symptomatology over time. We infer that this change may be explained by the fact that having had a chronic disease for a long time without experiencing acute or severe events, including COVID-19 infection, might have led these patients to perceive their health condition as less severe^28^ contributing to a more positive emotional state.

Informative and significant associations were found. Two impact analyses were conducted shedding light on the longitudinal prediction of depression symptoms on the psychological response to the pandemic. The first analysis showed that higher levels of baseline depression symptoms significantly contributed to increasing psychological distress scores after 10 years. To our knowledge, no similar longitudinal findings have been reported so far. As for the role of anxiety, although a positive correlation was found, no significant prediction emerged, revealing a weaker effect of anxiety when compared to the simultaneous impact of depression. This effect might be explained by the fact that patients were interviewed immediately after the first lockdown and, thus, in the context of quarantine. Accordingly, studies on older adults show predominantly higher incidence of depressive symptoms when they are forced to stay home and consequently reduce social interactions^5,29^. Moreover, most did not work or were retired, being less exposed to direct contact with others and, thus, to potential contagion that could have generated fear and anxiety. Consistently, it was shown that more direct exposure to pandemic illness threats are mainly associated with anxiety and worry and that excessive responses can be debilitating and lead to maladaptive behaviours (e.g., extensive washing and cleaning, compulsive hand sanitizing)^30^.

Living conditions are the only socio-demographic factor that had a significant impact, meaning that living with others significantly lowered psychological distress. This association is in line with prior works concerning the impact of social isolation on the mental health of older people due to COVID-19^31^. Although the preventive measures adopted during the pandemic have constrained older people from social participation, limiting its protective influence on diverse health-related domains (e.g., disability, quality of life, cognition)^32^, living with others may have increased perceived social support mitigating the effect of psychological distress^33^. Consistently, a recent study showed that, during the quarantine, social support was negatively associated with irritability, insomnia, and anxiety and that higher levels reduced the risk of depression^34^.

The second regression analysis provided evidence in support of the effect of depression symptoms on the psychological impact of COVID-19 over time. Not only was baseline depression symptom prediction confirmed, but also the change in symptoms after 10 years emerged as a significant independent factor. The reduction of depression symptoms over ten years seemed to predict lower levels of psychological distress, corroborating the idea that better mental health and higher psychological well-being may represent protective factors against negative psychological responses to the pandemic^35^. Again, baseline anxiety symptoms seemed not to have a significant impact, nor the change over time. As mentioned before, if we consider that participants experienced social interaction reduction due to quarantine, and had no direct contact with the virus we infer that depression symptomatology, in this case, may have had a larger effect on psychological distress than anxiety making it a predominant predisposing factor.

While this study provides new insights on the predictors of COVID-19-related psychological distress, it has some limitations. The sample size was modest and mostly included male patients aged over 60 and affected by a specific chronic non-communicable disease. Although it provides reliable and valid insights into this population segment, it limits the generalizability of the results. To better target the psychological impact of the COVID-19 pandemic on a frail population, future studies should also consider depression and anxiety symptoms in relation to other chronic conditions (e.g., respiratory diseases), whose severity would critically complicate COVID-19 progression, and larger sample sizes. Moreover, it must be underscored that this study adopted a self-report method. Even though these types of studies may be constrained by methodological and inferential limitations (e.g., social desirability bias), they are suitable to provide informative insights into a phenomenon^36^. Moreover, they have some advantages, including high practicality of use, clinical and research applicability, and good cost-effectiveness. Accordingly, it has been recently shown that self-reported behaviors and observations of actual behaviors during the COVID-19 pandemic overlap^37^. Lastly, it must be noted that the current study was carried out in a specific timeframe and context. Recognizing that the extent and the severity of the pandemic varied drastically among countries, it is essential to consider and interpret the present findings cautiously with regards to their generalizability.

Despite its limitations, this study presents relevant strengths. Firstly, our findings provide precious evidence on the impact of pre-existing conditions on the psychological impact of the pandemic representing an added value for the existing literature on the associations among depression, anxiety, and psychological distress. Specifically, it is noteworthy that the adoption of a longitudinal design made it possible to observe the impact within a timeframe of ten years, which, to our knowledge, represents a unique confirming result. Secondly, the assessment timeframe was immediately after the first pandemic peak, and this allowed us to collect data during one of the most psychologically challenging periods so far. Furthermore, differently from most pandemic-focused studies which conducted online questionnaire surveys, this study was carried out through telephone interviews. The use of structured interviews allowed us to better reach a segment of the population that would have found online surveys more difficult to handle due to old age and unfamiliarity with the internet.

To sum up, results provide insight into the impact of pre-existing conditions on the psychological response to COVID-19 pandemic. Depression symptoms and their change over time significantly predicted the psychological impact of pandemic 10 years later shedding light on their longitudinal predisposing influence. Additionally, living with others revealed to be a protective factor contributing to mitigate the levels of perceived psychological distress. Besides, these findings have crucial clinical and health-related implications. Monitoring mood-specific risk factors and the living condition among vulnerable populations, namely older people with chronic diseases, should be a paramount concern during the pandemic era. Prevention approaches and interventions that take the influence of such factors into consideration are needed in order to put in place public health strategies aimed at pre-empting the severe mental health consequences of COVID-19 and, consequently, improving the health-related quality of life of these clinical populations.

## Methods

### Study design, participants, and procedure

The current investigation is a follow-up survey of a longitudinal study (started in February 2011) involving 345 patients with essential arterial hypertension^38–40^. Patients who met the inclusion criteria of the study were aged between 30 and 75, diagnosed with essential arterial hypertension, receiving regular pharmacological treatment, with sufficient Italian language skills, and with no cognitive deficits or concomitant major pathologies (e.g., cancer). All participants were recruited at the same hospital and underwent a longitudinal evaluation consisting of repeated standardized measures and questionnaires aiming to profile their psychological, behavioral, and clinical characteristics.

For this follow-up, telephone data collection was conducted through a structured interview within 2 months immediately after the first lockdown in Italy (May–August 2020). According to the epidemiological data confirmed by the WHO Health Emergency Dashboard, the timeframe considered refers to the first contagion peak in Italy^2^. Of the original sample, patients currently aged over 60 (n = 232) were contacted. The choice to recruit older participants with a chronic condition was driven by the evidence that this population is more likely at risk of developing severe forms of COVID-19^41,42^. Of the 232 selected patients, 104 were excluded because they did not answer the call. Of the remaining 128 contacts, 23 refused to join the study, for a total drop-out in the current follow-up of 127 patients (54.7%). These differed significantly by age, being older on average (72.1 ± 6.9), than the participating patients (69.6 ± 5.8; p < 0.01). Moreover, a significant difference was found in baseline depression symptoms, which were significantly higher in the non-participating patients (5.7 ± 3.4) than in those who joined this study (4.3 ± 3.0; p < 0.001). No significant differences in gender, time since hypertension diagnosis, and baseline mean scores of anxiety symptoms were found. Table 5 shows the socio-demographic characteristics of the final sample (n = 105). The participants had a mean age of 69.6 ± 5.8 and were primarily male (60.6%). The proportion of men in the sample reflects the cardiovascular disease incidence, which is more common among men than women^43^. The majority lived with others (87.5%), did not work or were retired (73.1%). Moreover, most had suffered from hypertension for more than ten years (76.0%).

**Table 5:** Socio-demographic characteristics of the study sample.

Socio-demographic variables	
Age (years)	69.6 ± 5.8
**Gender**	
Male	63(60.6%)
Female	41(39.4%)
Living conditions	
Alone	13(12.5%)
With others	91(87.5%)
**Employment status**	
Working	28(26.9%)
Not working or retired	76(73.1%)
**Time since hypertension diagnosis**	
Less than 10 years	23(24.0%)
10 years or more	73(76.0%)

Data are mean ± standard deviation or %.

Both the larger longitudinal study and this follow-up survey were approved by the Ethics Committee of the University of Milano-Bicocca. At baseline, informed consent allowing for future contact through the longitudinal study was obtained. For the current follow-up, participants received written information about the purpose of the study and voluntarily signed a consent form to participate. The present research was conducted in accordance with the Declaration of Helsinki and all relevant guidelines and regulations.

### Measures

The structured telephone interviews were conducted by two of the authors with a background in psychology and prior experience in clinical research. Notably, preliminary planned discussions with all authors were undertaken to acquire the necessary information and expertise to optimally conduct data collection and pilot trainings were carried out to increase the familiarity with the structure of the interview. On average, the interviews lasted 30 min. They were comprised of 1) a preliminary section aimed at collecting participants’ updated socio-demographic characteristics as well as general and COVID-19-related health indicators, and 2) a section composed of standardized questionnaires measuring depression and anxiety symptoms, and the psychological impact of COVID-19 pandemic.

### Socio-demographic and health-related indicators

Socio-demographic information included living conditions (alone vs. with others) and employment status (working vs. not working/retired). Age, gender, and time since hypertension diagnosis were known a priori and, thus, were not asked. The following questions concerned general health status and referred to the clinical condition. Patients were also asked whether they or their relatives had contracted COVID-19. If the answer was ‘yes’, patients were asked information about the clinical progression (e.g., severity of symptoms, hospitalization).

### Anxiety and depression

To ensure consistency and accurate comparisons with prior data collections, anxiety and depression symptoms evaluation was conducted with the Hospital Anxiety and Depression Scale (HADS)^44^. This is a 14-item self-report questionnaire measuring anxiety and depression levels in medical patients. Sample items for the anxiety factor are: “I get a sort of frightened feeling as if something awful is about to happen”, “Worrying thoughts go through my mind”; and for the depression factor: “I have lost interest in my appearance”, “I feel as if I am slowed down”. The anxiety and depression subscales are evaluated with 7 items scored on a 4-point Likert scale (range, 0–21). Higher scores indicate increased severity of symptomatology. The Italian validation^45^ determined cut-offs for symptom severity as follows: the absence of symptoms (≤ 7), mild (8–10), moderate (11–14), and severe (≥ 15) symptoms, and showed high internal consistency for both subscales (Anxiety, α = 0.85; Depression, α = 0.84). Again, for the present study satisfactory levels were observed (Anxiety, α = 0.77; Depression, α = 0.80).

### Psychological impact of COVID-19 pandemic

The psychological impact of the COVID-19 pandemic was evaluated with the Impact of Event Scale-Revised (IES-R)^46^. So far, the IES-R has been widely used in prior studies on the psychological effects of COVID-19 and has shown satisfactory results^26^. It is a validated 22-item self-report questionnaire scored on a 5-point Likert scale measuring perceived stress caused by a traumatic event. It comprises 3 subscales (i.e., Intrusion, Avoidance, and Hyperarousal) which are closely affiliated with post-traumatic stress disorder (PTSD) symptoms. Higher scores reflect higher levels of symptoms. Patients were asked to rate their level of distress referring to their perceived emotional state towards COVID-19. A specification of the items was performed by the research group in order to better refer to the COVID-19 pandemic as the event evaluated. Sample items are: “My feelings about COVID-19 were kind of numb”, “Any reminder brought back feelings about COVID-19”. The total IES-R score was categorised for severity as follows: normal (≤ 23), mild (24–32), moderate (33–36), and severe psychological impact (≥ 37). According to the original validation, a cut-off score of 24 was considered as defining PTSD^47^. The Italian validation^48^ showed satisfactory internal consistency for all subscales (Intrusion, α = 0.78; Avoidance, α = 0.72; Hyperarousal, α = 0.83). Likewise, in the present study, overall high reliability levels were observed (α = 0.87).

### Statistical analyses

Descriptive statistics on socio-demographic and psychological characteristics of the sample were calculated. Mean and standard deviation (SD) for continuous variables and percentages for categorical variables were reported. Data normal distribution was tested by calculating skewness and kurtosis indices, and respective recommended ranges of ± 2 and ± 7 were considered for normality^49^. To describe the degree of the longitudinal change of anxiety and depression symptoms, a delta was calculated considering the baseline and the current follow-up mean scores. Then, a paired-samples *t*-test was conducted to detect significant mean differences. A correlational analysis was also performed to explore the associations among depression, anxiety and psychological distress. Two multiple linear regression models were analysed. The first one included the socio-demographic characteristics (i.e., age, gender, occupation, living conditions, and time since hypertension diagnosis), and anxiety and depression (HADS subscales) measured at baseline as independent variables. The overall score of the psychological impact of COVID-19 (IES-R) was defined as a dependent variable. In the second one, the same structure was defined with the addition of anxiety and depression computed deltas as further predictors. For both models, adjusted *R*^2^ and *F* test values were calculated for the explained variance and model fit, respectively. The analyses were conducted by means of the Statistical Package for Social Sciences (SPSS) software version 26.0. All statistical tests were two-tailed and a p-value ≤ 0.05 was considered statistically significant (Suppl. Information).

## Supplementary Information

Supplementary Tables.

## Data Availability

The datasets generated and/or analysed during the current study are not publicly available due to privacy or ethical restrictions but are available from the corresponding author on reasonable request.
