# Supplementary material for: Depression symptoms as longitudinal predictors of the psychological impact of COVID-19 pandemic in hypertensive patients
Source: Sci Rep. 2021 Aug 13;11:16496. doi: 10.1038/s41598-021-96165-2 (PMC8363734; doi:10.1038/s41598-021-96165-2)
Supplement: Supplementary file 1 — Supplementary Tables. [file 41598_2021_96165_MOESM1_ESM.pdf]

Appendix. Statistical Analyses

**Variable**	**Non-participants (n=127, 54.7%)**	**Participants (n=105, 45.3%)**	***t***	**χ^2**	***P* value**
Age (years)	72.1±6.9	69.5±5.8	3.047		.003
Gender				.975	.353
Male	68(53.5%)	63(60.0%)			
Female	59(46.5%)	42(40.0%)			
Time since hypertension diagnosis				.509	.503
Less than 10 years	22(19.6%)	23(23.7%)			
10 years or more	90(80.4%)	74(76.3%)			
HADS					
Anxiety (baseline)	7.7±4.1	7.1±3.7	1.280		.202
Depression (baseline)	5.7±3.3	4.3±3.0	3.541		<.001

Groups comparisons between non-participating patients and participating patients for age, gender, time since hypertension diagnosis, and baseline anxiety and depression symptoms. Unpaired-samples t-test (*t*) and chi-squared (χ^2) test were performed for continuous and categorical variables, respectively. HADS, Hospital Anxiety Depression Scale.

**Variable**	**Skewness**		**Kurtosis**	
	**Estimate**	**SE**	**Estimate**	**SE**
HADS				
Anxiety (baseline)	.565	.236	.003	.467
Depression (baseline)	.574	.236	-.448	.467
Anxiety (follow-up)	.975	.236	.315	.467
Depression (follow-up)	1.601	.236	2.199	.467
IES-R	1.226	.237	1.604	.469

Results from normal distribution analysis. Normality ranges of ±2 and ±7 were considered for skewness and kurtosis, respectively. Estimates and standard errors (SE) are reported for each variable. HADS, Hospital Anxiety Depression Scale. IES-R, Impact of Event Scale-Revised.

	**1**	**2**	**3**	**4**	**5**
**1. Anxiety (baseline)**	-				
**2. Depression (baseline)**	.610**	-			
**3. Anxiety (follow-up)**	.418**	.352**	-		
**4. Depression (follow-up)**	.291*	.514**	.505**	-	
**5. IES-R**	.317*	.373**	.271*	.476**	-

Correlations among baseline and follow-up anxiety and depression and pandemic-related psychological distress.
* p value <.01, ** p value <.001
IES-R, Impact of Event Scale-Revised

Model	R	R Square	Adjusted R Square	Std Error of the Estimate
1	.519	.269	.211	11.49

Model 1 summary.
Dependent Variable: pandemic-related psychological distress (IES-R)
Predictors: age, gender, living condition, employment status, time since hypertension diagnosis, baseline anxiety and depression

Model		Sum of Squares	Degree of Freedom	Mean Square	*F*	*P* value
1	Regression	4279.714	7	611.388	4.630	<.001
	Residual	11621.244	88	132.060		
	Total	159000.958	95			

ANOVA, Model 1 fit.
Dependent Variable: pandemic-related psychological distress (IES-R)
Predictors: age, gender, living condition, employment status, time since hypertension diagnosis, baseline anxiety and depression

	β	*t*	95% CI		*P* value
Age	-.246	-1.175	-.662	.170	.243
Gender	-.035	-.014	-4.902	4.833	.989
Living conditions	-7.274	-2.072	-14.250	-.297	.041
Employment status	4.920	1.769	-.606	10.446	.080
Time since hypertension diagnosis	-1.313	-.460	-6.990	4.363	.647
HADS Anxiety (baseline)	.492	1.202	-.321	1.306	.232
HADS Depression (baseline)	1.483	2.905	.468	2.498	.005

Model 1. Multiple linear impact of socio-demographic variables, and baseline anxiety and depression symptoms on IES-R (n=96). Data are the unstandardized regression coefficients (*β*), the t-test value (*t*), and confidence interval (95%)

Model	R	R Square	Adjusted R Square	Std Error of the Estimate
2	.633	.400	.338	10.52

Model 2 summary.
Dependent Variable: pandemic-related psychological distress (IES-R)
Predictors: age, gender, living condition, employment status, time since hypertension diagnosis, baseline anxiety and depression, anxiety and depression change after 10 years (delta)

Model		Sum of Squares	Degree of Freedom	Mean Square	*F*	*P* value
2	Regression	6367.075	9	707.453	6.382	<.001
	Residual	9533.884	86	110.859		
	Total	15900.958	95			

ANOVA, Model 2 fit.
Dependent Variable: pandemic-related psychological distress (IES-R)
Predictors: age, gender, living condition, employment status, time since hypertension diagnosis, baseline anxiety and depression, anxiety and depression change after 10 years (delta)

	β	*t*	95% CI		*P* value
Age	-.277	-1.422	-.665	.111	.159
Gender	1.036	.457	-3.468	5.540	.649
Living conditions	-3.971	-1.169	-10.722	2.780	.245
Employment status	2.526	.969	-2.657	7.709	.335
Time since hypertension diagnosis	-1.043	-.398	-6.248	4.161	.691
HADS Anxiety (baseline)	.579	1.270	-.327	1.486	.207
HADS Depression (baseline)	2.261	4.373	1.233	3.289	<.001
HADS Anxiety (Δ)	-.151	-.385	-.931	.629	.701
HADS Depression (Δ)	-1.640	-3.765	-2.506	-.774	<.001

Model 2. Multiple linear impact of socio-demographic variables, baseline anxiety and depression symptoms, and their change after 10 years on IES-R (n=96). Data are the unstandardized regression coefficients (*β*), the t-test value (*t*), and confidence interval (95%).
